# Constructal blade shape in nanofluids

**DOI:** 10.1186/1556-276X-6-240

**Published:** 2011-03-21

**Authors:** Chao Bai, Liqiu Wang

**Affiliations:** 1 Department of Mechanical Engineering, The University of Hong Kong, Pokfulam Road, Hong Kong

## Abstract

Blade configuration of nanofluids has been proven to perform much better than dispersed configuration for some heat conduction systems. The analytical analysis and numerical calculation are made for the cylinder--shaped and regular-rectangular-prism--shaped building blocks of the blade-configured heat conduction systems (using nanofluids as the heat conduction media) to find the optimal cross-sectional shape for the nanoparticle blade under the same composing materials, composition ratio, volumetric heat generation rate, and total building block volume. The regular-triangular-prism--shaped blade has been proven to perform better than all the other three kinds of blades, namely, the regular-rectangular-prism--shaped blade, the regular-hexagonal-prism--shaped blade, and the cylinder--shaped blade. Thus, the regular-triangular-prism--shaped blade is selected as the optimally shaped blade for the two kinds of building blocks that are considered in this study. It is also proven that the constructal cylinder--regular-triangular-prism building block performs better than the constructal regular-rectangular-prism--regular-triangular-prism building block.

## Introduction

Nanofluids are mixtures of nanoparticles and base fluids, which have different thermal conductivities [1-5]. They were identified and proposed as a result of people's persistent pursuit for more and more efficient heat-transfer media. It should be noted that conventional heat transfer fluids have normally very low thermal conductivity, thus destroying much exergy during heat transport. At present, a great amount of attention is paid to studies on nanofluids, with the aim of addressing many unsolved issues [6-10].

Constructal theory is a novel thought for nature and society [11-17]. It tries to explain phenomena based on optimization, or natural selection in biological terms. One of its viewpoints is that two flow mechanisms are better than one [18]. This is what one sees naturally in river basins, lung structure, and the percolation threshold effect [19]. Our previous studies have proved that the blade configuration of nanofluids is much better than the dispersed configuration for the two kinds of disk-shaped heat conduction systems with different boundary conditions [20,21]. It is believed that the continuous nanoparticle blades with higher thermal conductivity serve as the second conduction mechanism, and its optimized cooperation with the base fluid of low thermal conductivity leads to the much better performance. In this study, the blade configuration of nanofluids is considered in detail by studying the influence of the shapes of high-conductivity blades in two kinds of building blocks of the total blade-configured heat conduction systems. This study is inspired by the need for the optimization of the cross section of duct for minimum flow resistance [11]. By treating heat as a flow medium flowing in blades, one can also find the optimal shape for the blades, which offers minimum thermal resistance.

## Optimal blade shape for two kinds of building blocks of blade-configured heat conduction systems

**Figure 1:**
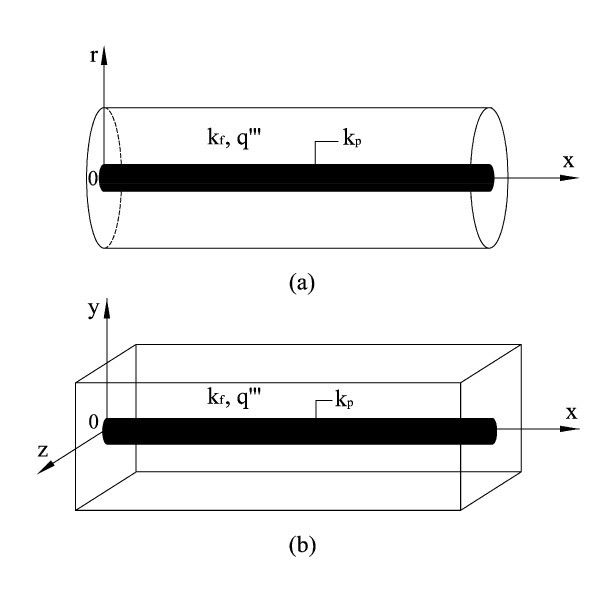
**Two kinds of heat conduction building blocks considered in this study: **(a) **cylinder--cylinder building block; **(b) **rectangular-prism--cylinder building block**.

The cylinder--shaped and regular-rectangular-prism--shaped building blocks are studied in this article. The nanoparticle blade has four different shapes: regular triangular prism, regular rectangular prism, regular hexagonal prism, and cylinder. For conciseness, the word "regular" is omitted hereafter. A format of "building-block-shape--blade-shape" is used to indicate the eight kinds of building blocks. Figure 1 shows the two kinds of building blocks with cylinder blades. For all the eight kinds of building blocks, uniform heat generation rate occurs in the base fluid region. All the external surfaces, except the cross-sectional plane *x *= 0 of the blade, are adiabatic. The cross-sectional plane *x *= 0 of the blade serves as the heat sink with constant temperature. The composition of these two-material building blocks is fixed by volume fraction(1)

It is assumed that *ϕ *≪ 1, and the thermal conductivity ratio of nanoparticle material and base fluid material is fixed and large. The thermal contact resistance is not considered.

In order to study the influence of the blade shape, the materials of the base fluid and nanoparticle, volumetric heat generation rate, and volumes of the eight kinds of building blocks are also fixed, besides the volume fraction and thermal conductivity ratio; however, the slenderness is free to vary to achieve the constructal system (building block) overall temperature difference. Here, the slenderness refers to the ratio of the radius to length for the cylinder building block, and the ratio of the circumscribing cylinder radius to length for the rectangular prism building block. For the simplest cylinder--cylinder building block, analytical analysis can be made; the system overall temperature difference, the constructal system overall temperature difference, and the constructal slenderness can be obtained analytically. Based on a slenderness range predicted by the analytic result, the numerical calculation is then conducted for all the eight kinds of systems to obtain, as accurately as possible, the results for comparison among different blade shapes and different building block shapes.

### Analytical analysis for cylinder--cylinder building block

Owing to the much higher thermal conductivity of nanoparticle material, heat conduction inside this kind of building block can be considered to consist of two one-dimensional routes-radial conduction inside cross-sectional planes of the base fluid region and axial conduction along the blade.

For the radial heat conduction inside base fluid, the governing equation and boundary conditions are(2)

and(3)

respectively, where, *k*_f _is the thermal conductivity of the base fluid; q''' is the volumetric heat generation rate; *T*_c _is the temperature at the interface of the blade and base fluid at *x *= *L*_0 _(*L*_0 _is the length of the building block); *r*_0 _is the radius of the inner blade; and *R*_0 _is the outer radius of the building block. By solving Equations (2) and (3), the radial temperature distribution of the cross-sectional plane *x *= *L*_0 _of the base fluid region is obtained:(4)

For the axial conduction inside the blade, the governing equation and boundary conditions, respectively, are(5)

and(6)

where *k*_p _is the thermal conductivity of the nanoparticle material, and *T*_0 _is the heat-sink temperature at the cross-sectional plane *x *= 0 of the blade. By solving Equations (5) and (6), the axial temperature distribution along the blade is obtained as(7)

Thus, the overall temperature difference of the building block is(8)

Nondimensionalizing this overall temperature difference with  (constant), one has(9)

**Figure 2:**
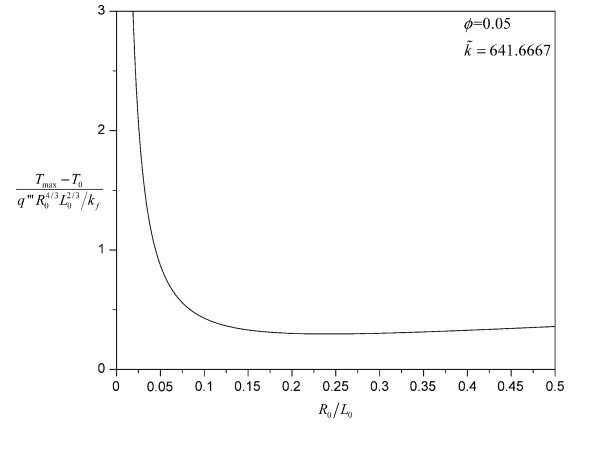
**Variation of the nondimensional system overall temperature difference with slenderness for cylinder--cylinder building block (analytical result, *ϕ *= 0.05 and  = 641.6667)**.

Where  is the nondimensional system (building block) overall temperature difference, and  is the ratio of the thermal conductivities of the nanoparticle material and the base fluid material. By substituting Equation (1) in Equation (9),  becomes(10)

which indicates that the building block's overall temperature difference (or thermal resistance) depends on its slenderness  under the same composing materials, composition ratio, volumetric heat generation rate, and total volume. Figure 2 typifies this dependence at *ϕ *= 0.05 and  = 641.6667 (thermal conductivity ratio of copper and water). By minimizing this nondimensional system overall temperature difference with respect to , the nondimensional constructal system overall temperature difference can be obtained:(11)

and(12)

At , the best-performing cylinder--cylinder building block can be obtained. If one specifies *ϕ *= 0.05 and  = 641.6667, then the optimal slenderness  will be 0.240618, and the nondimensional constructal system overall temperature difference  will be 0.296778, which is the lowest point in Figure 2. Similarly, for the other kinds of building blocks considered here, there also exists such a best-performing slenderness .

### Numerical calculation for all the eight kinds of building blocks

The actual heat conduction in the cylinder--cylinder building block is of course not a simple combination of two one-dimensional conductions. For the other kinds of building blocks, the flow of heat is even more complex. In order to have as accurately as possible results for the comparison, a finite volume computational fluid dynamics (CFD) code [22] is used for obtaining numerical results for all the eight kinds of building blocks.

The conservation of energy equations are(13)

and(14)

for the base fluid and the blade, respectively. Here,(15)

For rectangular-prism--series building blocks, *R*_0 _stands for the radius of the circumscribing cylinder, for which one has  (where *a *is the side length of the rectangular cross section). Constant, , is introduced to use fewer grids to achieve accurate enough results. The "+1" in the nondimensional temperature expression is introduced to ensure that the computing process does not touch the limit of 0 K. At the interface between the *k*_f _region and *k*_p _blade, the continuity of heat flux requires that(16)

where **ñ **is the nondimensional normal vector; . As all the external surfaces, except the plane *x *= 0 of the *k*_p _blade, are adiabatic,(17)

and for the plane *x *= 0 of the *k*_p _blade, one has(18)

Note that  is exactly the nondimensional system overall temperature difference, , as shown in Equation (9), where  is the maximal nondimensional temperature in the heat conduction building blocks.

It is specified that *ϕ *= 0.05 and  = 641.6667 for the numerical calculation, the value used in the analytical analysis. The finite volume CFD code is chosen because of its efficiency and flexibility to generate a large number of results for various geometries which differ slightly from each other. Hexahedron grids are used to mesh the cylinder--hexagonal-prism and cylinder--cylinder building blocks, whereas all the other building blocks are meshed with wedge grids. Appropriate grid number is determined by doubling the interval number in , , and  directions each time, until the change of temperature becomes less than 0.05% (the maximal temperature, , is used specifically for this criterion). Table 1 shows an example of how this grid independence is reached.

**Table 1:** Grid-independence check (cylinder--triangular-prism building block, R_0_/L_0 _= 0.25)

Number of grids		**Changing of **
2500	0.271978	-0.010181 × 10^0^
20120	0.269209	-8.803569 × 10^-4^
167360	0.268972	-3.346073 × 10^-5^
1248160	0.268963	

**Figure 3:**
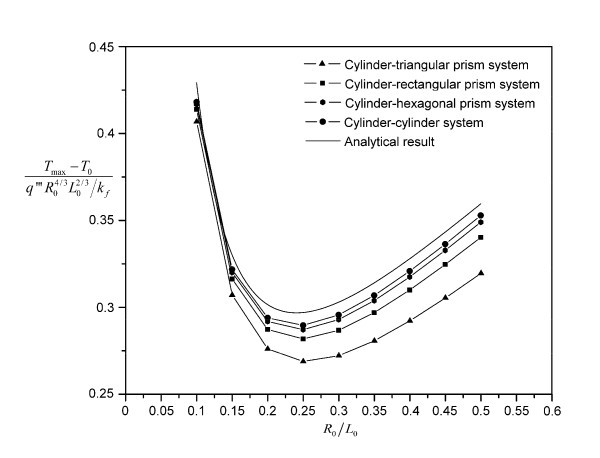
**Numerical results for the cylinder--series building blocks and the analytical result for the cylinder--cylinder building block (*ϕ *= 0.05 and  = 641.6667)**.

**Figure 4:**
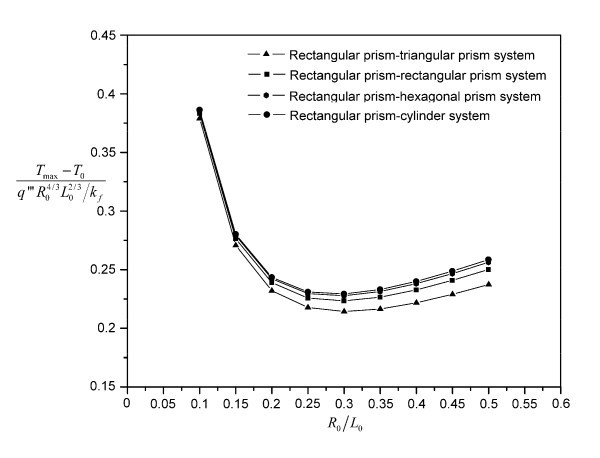
**Numerical results for the rectangular-prism--series building blocks (*ϕ *= 0.05 and  = 641.6667)**.

The nondimensional system overall temperature difference is shown in Figures 3 and 4 for the eight kinds of building blocks. The optimal slenderness  and nondimensional constructal system overall temperature difference  for the cylinder--cylinder building block are 0.25 and 0.289577, respectively. Comparing with the approximate analytical results, the differences are only 3.75% and 2.49%, which confirms the accuracy of the finite volume CFD code.

All the eight kinds of heat conduction building blocks show strong performance dependence on the slenderness  of the building blocks. Under the specific values for *ϕ *and , the optimal slendernesses for the cylinder--series and rectangular-prism--series building blocks are 0.25 and 0.3, respectively, within a resolution of 0.05. Below the optimal slenderness, the performance dependence on  increases in the order from cylinder--cylinder building block to cylinder--hexagonal-prism building block, cylinder--rectangular-prism building block, and then cylinder--triangular-prism building block. Above the optimal slenderness, this trend changes its direction. A similar situation happens for the rectangular-prism--series building blocks. When the  value is small enough ( for both the cylinder--series and rectangular-prism--series building blocks), the blade shape begins to have very weak effect on the system's performance, due to the diminishing role played by the building block's cross-sectional area when compared to its length. It can be seen from both Figures 3 and 4 that the four nondimensional system overall temperature difference curves almost collapse into one curve. For the cylinder--series building blocks, the collapsing curves approach the analytical result of the cylinder--cylinder building block as expected since the analytical result becomes more and more accurate as the  value decreases. The analytical result shown in Figure 3 has the same *ϕ *and  values as those for the numerical calculation (the same curve as Figure 2). Thus, the accuracy of the finite volume CFD code is verified again. It should be noted that both the cylinder--series and rectangular-prism--series building blocks have fixed optimal slenderness. Therefore, the triangular-prism--shaped blade always performs the best among the four kinds of blades considered. Besides, the performance difference between successively shaped blades decreases from triangular-prism blade to cylinder blade, which is consistent with their surface area changing under the same volume, as shown in Table 2. Here, the surface area of the blade is exactly the interfacial area between the base fluid and the blade. Thus, it is very likely that the performance difference can be attributed to the interfacial area difference: larger interfacial area will facilitate more low-temperature surfaces for the heat generation region, thereby lowering the maximal temperature in that region.

**Table 2:** Perimeters of four blade cross sections having the same area of *π*

Cross section shape	Perimeter
Triangle	= 8.080642
Rectangle	= 7.089816
Hexagon	= 6.597817
Circle	2*π *= 6.283186

Therefore, under the same composing materials ( = 641.6667), composition ratio (*ϕ *= 0.05), volumetric heat generation rate and total volume, the cylinder--triangular-prism and rectangular-prism--triangular-prism building blocks with slenderness values of 0.25 and 0.3, respectively, should be used for achieving the lowest system overall temperature difference (or, system thermal resistance) in practical applications. Both the pursuits of energy and material savings make this aim very significant. Furthermore, if one can also set the outer shape of the heat conduction building block free (often constrained by efficient packing and manufacturing), a comparison can be made between the constructal (both the blade shape and slenderness having been optimized) cylinder--triangular-prism building block and constructal rectangular-prism--triangular-prism building block. Since the total volumes for the cylinder--series and rectangular-prism--series building blocks are  and , respectively, to ensure that the comparison is based on the same total building-block volume, the nondimensional constructal system overall temperature difference of the cylinder--triangular-prism building block is divided by *π*^2/3^(19)

and the nondimensional constructal system overall temperature difference of the rectangular-prism--triangular-prism building block is divided by 2^2/3^(20)

Thus, the constructal cylinder--shaped heat conduction building block performs better than the rectangular-prism--shaped building block.

## Conclusions

Inspired by the duct cross section optimization for minimum flow resistance, the shape of the nanoparticle blade is optimized for the cylinder--shaped and rectangular-prism--shaped building blocks of the blade-configured heat conduction systems (blade configuration of nanofluids) based on the same composing materials, composition ratio, volumetric heat generation rate, and total building block volume. The four kinds of blade shapes are triangular prism, rectangular prism, hexagonal prism, and cylinder. For the cylinder--cylinder building block, analytical analysis can be conducted. Explicit expressions for the system overall temperature difference, constructal system overall temperature difference, and constructal slenderness can be obtained. Then, based on the slenderness range predicted by the analytical result, numerical calculations are performed for the eight kinds of building blocks to obtain as accurately as possible results for comparison. One specifies that *ϕ *= 0.05 and  = 641.6667 for the numerical calculation.

The performances of the eight kinds of building blocks depend strongly on the building-block slenderness. The constructal slendernesses leading to minimum system overall temperature differences (system thermal resistances) are 0.25 and 0.3, respectively, for the cylinder--series and rectangular-prism--series building blocks. For both the cylinder--series and rectangular-prism--series building blocks, the triangular-prism--shaped blade performs the best among all the four kinds of blades considered. This is explained by the size of interfacial area sustained by the four kinds of blades with a fixed volume. Also, the constructal cylinder--triangular-prism building block is proved to perform better than the constructal rectangular-prism--triangular-prism building block at the same composing materials, composition ratio, volumetric heat generation rate and total building-block volume.

## Abbreviations

CFD: computational fluid dynamics.

## Competing interests

The authors declare that they have no competing interests.

## Authors' contributions

Both authors contributed equally.
